# Simultaneous Characterization of Two Ultrashort Optical Pulses at Different Frequencies Using a WS_2_ Monolayer

**DOI:** 10.1021/acsphotonics.1c01270

**Published:** 2022-05-10

**Authors:** Marcus L Noordam, Javier Hernandez-Rueda, L. Kuipers

**Affiliations:** Kavli Institute of Nanoscience Delft, Department of Quantum Nanoscience, Delft University of Technology, Lorentzweg 1, 2628 CJ Delft, The Netherlands

**Keywords:** FROG, four-wave mixing, sum-frequency generation, ultrashort laser pulses, 2D materials, double-blind pulse characterization

## Abstract

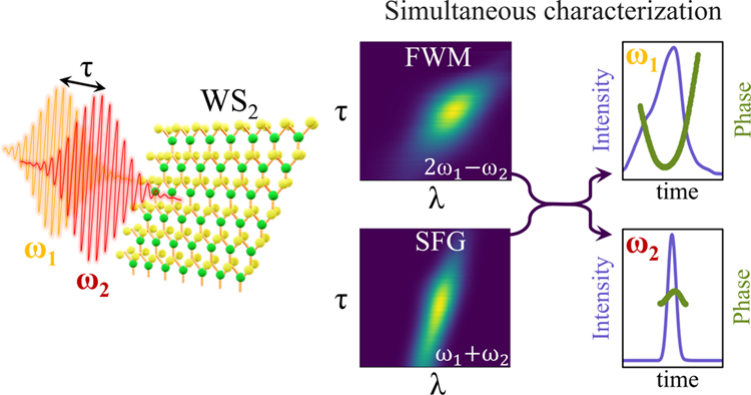

The precise characterization of ultrashort laser pulses has been of interest to the scientific community for many years. Frequency-resolved optical gating (FROG) has been extensively used to retrieve the temporal and spectral field distributions of ultrashort laser pulses. In this work, we exploit the high, broad-band nonlinear optical response of a WS_2_ monolayer to simultaneously characterize two ultrashort laser pulses with different frequencies. The relaxed phase-matching conditions in a WS_2_ monolayer enable the simultaneous acquisition of the spectra resulting from both four-wave mixing (FWM) and sum-frequency generation (SFG) nonlinear processes while varying the time delay between the two ultrashort pulses. Next, we introduce an adjusted double-blind FROG algorithm, based on iterative fast Fourier transforms between two FROG traces, to extract the intensity distribution and phase of two ultrashort pulses from the combination of their FWM and SFG FROG traces. Using this algorithm, we find an agreement between the computed and observed FROG traces for both the FWM and SFG processes. Exploiting the broad-band nonlinear response of a WS_2_ monolayer, we additionally characterize one of the pulses using a second-harmonic generation (SHG) FROG trace to validate the pulse shapes extracted from the combination of the FWM and SFG FROG traces. The retrieved pulse shape from the SHG FROG agrees well with the pulse shape retrieved from our nondegenerate cross-correlation FROG measurement. In addition to the nonlinear parametric processes, we also observe a nonlinearly generated photoluminescence (PL) signal emitted from the WS_2_ monolayer. Because of its nonlinear origin, the PL signal can also be used to obtain complementary autocorrelation and cross-correlation traces.

## Introduction

Since the realization of ultrashort laser pulses, there has been much interest in accurately retrieving their temporal intensity distribution. Ultrashort laser systems readily produce pulses with a pulse duration that is too short to be directly measured with even the fastest photodiodes. Therefore, indirect autocorrelation methods are used to estimate the laser pulse duration, where the pulse interacts with itself and the time delay between two copies of the pulse is varied. However, autocorrelation methods intrinsically cannot provide the full pulse information, that is, spectral resolution is needed to retrieve the spectrum and time-dependent phase of the pulse.

One of the most investigated and commonly used techniques to characterize ultrashort laser pulses is frequency-resolved optical gating (FROG).^1,2^ In a FROG measurement, two ultrashort laser pulses are combined in a nonlinear medium. The spectrum of the nonlinear signal, generated via parametric optical processes, is recorded as a function of the time delay between the pulses, resulting in a FROG trace that contains both spectral and temporal information. FROG has been implemented using different optical setups and nonlinear processes including collinear setups, where the laser beam paths overlap, for second-harmonic generation (SHG),^3^ third-harmonic generation (THG),^4^ and noncollinear setups.^5^ In an autocorrelation FROG measurement, two copies of the same pulse are combined inside a nonlinear medium. Alternatively, in a cross-correlation FROG measurement, a known reference pulse is combined with an unknown pulse to generate nondegenerate signals such as sum-frequency generation (SFG) or four-wave mixing (FWM).^6−9^

To retrieve the pulse information of two unknown ultrashort pulses at different frequencies, the measurement of a FROG trace based on a single nondegenerate nonlinear process will not contain enough information. Therefore, cross-correlation measurements using nondegenerate nonlinear processes depend on a known reference pulse, a priori. With this method, a full characterization of two individual pulses still involves two measurements: an autocorrelation FROG measurement to retrieve the intensity distribution and phase of the reference pulse and the actual cross-correlation FROG measurement. The pulse shapes of two independent laser pulses can also be retrieved using multiple FROG traces based on different nondegenerate processes that are simultaneously measured.^10−12^

The most commonly used nonlinear media in FROG systems are nonlinear crystals due to their high nonlinear coefficients and broad frequency range of transparency. However, phase-matching requirements have to be satisfied in these nonlinear crystals, which can make experimental implementation difficult and restrict the frequency range of applicability, making simultaneous measurements of different nonlinear processes impractical. Nonlinear surface processes have a shorter interaction length and can overcome these phase-matching requirements. Therefore, nonlinear media with a large nonlinear surface response provide a route to generate and exploit multiple nonlinear processes simultaneously. The nonlinear surface response of plasmonic nanoantennas has been used for ultrashort pulse characterization.^12^ Atomically thin transition-metal dichalcogenide (TMDC) materials also show a remarkable high nonlinear surface response,^13−15^ along with an increase in absorption for photon energies above the semiconductor band gap.^16^ In addition to the background-free signals and the broad-band nonlinear response, TMDCs are also very promising materials for ultrashort pulse characterization due to their large atomically flat surface, which allows for easy beam alignment and transmission measurements. Since WS_2_ and related TMDC materials have large nonlinear susceptibilities over the whole visible wavelength range, they can be used to characterize laser pulses with a wide variety of wavelengths. Different types of TMDC monolayers or multilayer materials with different band-gap energies such as WSe_2_, MoS_2_, and MoSe_2_ could be selected depending on the wavelength of the ultrashort laser pulses and nonlinear processes employed to generate the FROG trace. Furthermore, different combinations of nonlinear FROG signals can be selected depending on the characterization demands at hand, specifically those related to the laser wavelengths.

Their fabrication process is relatively inexpensive and robust compared to plasmonic nanostructures. Nonlinear signals generated in WS_2_ could be further and selectively enhanced using more complex layered WS_2_ structures^17,18^ or combining WS_2_ with plasmonic nanostructures.^19^ Recently, FROG characterization of a single pulse using the SHG process has been performed on a WS_2_ monolayer.^20^

In addition to their atomic thickness, WS_2_ monolayers have another interesting property as nonlinear media that can be exploited for ultrashort pulse retrieval. The nonlinear generation of light-induced electron–hole pairs, the so-called excitons, can be potentially utilized in an autocorrelation measurement as its spectral width remains constant. The band structure of WS_2_ monolayers have a direct optical band gap at the K and K^–^ points allowing for the (nonlinear) generation of excitons.^21^ The radiative recombination of these excitons to the ground state can be observed as a photoluminescence (PL) signal.^15^

In this paper, we exploit the high nonlinear response of a WS_2_ monolayer over a broad spectral range to characterize two ultrashort laser pulses by measuring FROG traces based on the SHG, SFG, and FWM nonlinear processes. The spectra of the SFG and FWM processes are simultaneously recorded as a function of the time delay between two pulses with different wavelengths using a collinear optical setup. With a newly developed adaptation of a double-blind FROG algorithm, we precisely retrieve the pulse shape of two laser pulses at two different wavelengths, *E*_1_ and *E*_2_, using experimental FROG traces based on FWM and SFG that were simultaneously measured. We validate the pulse shape of one of the fundamental pulses using a separate SHG FROG measurement and by utilizing autocorrelation traces generated via nonlinear photoluminescence.

## Theory

To retrieve the complex pulse shapes *E*_1_ and *E*_2_ from nondegenerate FROG traces, we use an iterative retrieval algorithm based on the common pulse retrieval algorithm (COPRA), recently developed by Geib et al.^22^ The nonlinear process spectra of a measured noncollinear FROG trace can be defined as^23^
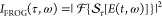
1Here, the frequency, ω, and the time delay, τ, are the parameters over which the measurement trace is evaluated. *E*(*t*,ω) is the electric field of the laser pulse,  is the signal operator that depends on the nonlinear process, and  is the Fourier transform operator. Although we use a collinear measurement scheme, the nondegenerate signals are background-free and thus collinear and noncollinear FROG traces are the same and therefore eq 1 still holds. The signal operators for the SFG (ω_SFG_ = ω_1_ + ω_2_) and FWM (ω_FWM_ = 2ω_1_ – ω_2_) processes as observed in our experiment, where the delay time of *E*_2_ is varied with respect to *E*_1_, can be derived as
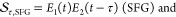
2
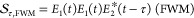
3The COPRA algorithm optimizes the pulses *E*_1_ and *E*_2_ so that the calculated FROG trace *I* matches the measured trace, *I*_meas_, as a nonlinear least-squares problem, further described in Geib et al.^22^ For a nondegenerate cross-correlation retrieval algorithm, one of the pulses is optimized, while the second pulse acts as a reference pulse. In this work, we modified the COPRA retrieval algorithm to optimize the pulse *E*_1_ or *E*_2_ for both experimental traces, *I*_meas,SFG_ and *I*_meas,FWM_, while the second pulse acts as the reference pulse, further described in the Supporting Information.

To simultaneously retrieve two unknown pulses *E*_1_ and *E*_2_, we utilize an iterative optimization scheme. Here, we start with two Gaussian pulses with central wavelengths λ_1_ and λ_2_ as our initial guess. Next, we start optimizing *E*_1_ for *I*_meas,SFG_ and *I*_meas,FWM_ while keeping *E*_2_ as a fixed reference pulse. Since an initial guess of *E*_2_ is used as a reference pulse, the pulse retrieval of *E*_1_ is not completed but the retrieved pulse shape of *E*_1_ is still a better approximation than the initial guess. In the next step, the newly retrieved approximation of *E*_1_ is used as a reference pulse in the retrieval of *E*_2_ resulting in a better approximation of *E*_2_. This process is iterated until the improvement of the normalized trace error between the measured and the retrieved FROG traces between iterations falls below a predefined value.

## Experimental Methods

**Figure 1:**
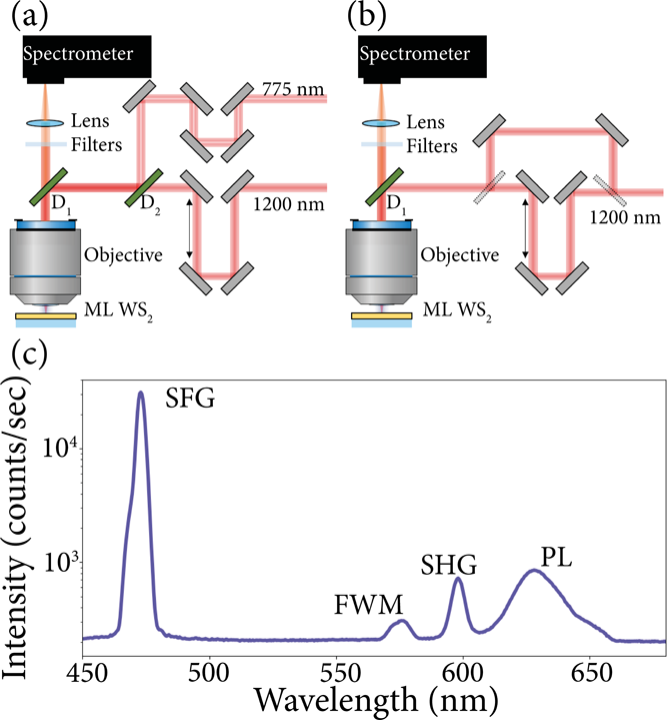
(a, b) Schematic representations of the optical setups. In panel (a), two laser pulses with wavelengths of 775 and 1200 nm are combined in a cross-correlation scheme to investigate nondegenerate nonlinear processes. In panel (b), two pulses with a wavelength of 1200 nm are combined in an autocorrelation scheme to investigate the degenerate nonlinear SHG process. (c) The measured nonlinear spectrum using the cross-correlation optical setup shown in panel (a) is plotted on a logarithmic scale.

In this work, we retrieve the pulse intensity distribution and phase of two femtosecond laser pulses with different central wavelengths. A femtosecond laser oscillator (Spectra-Physics Tsunami) generates pulses with a central wavelength of 775 nm. Part of this laser beam is used as our first laser pulse, while a fraction of it is used to pump an optical parametric oscillator (Spectra-Physics, Opal) that delivers the second laser pulse at 1200 nm. The pulse distributions of the 775 and 1200 nm laser pulses are retrieved by simultaneously measuring two cross-correlation FROG traces based on SFG and FWM signals using the optical setup sketched in Figure 1a. In addition to the nondegenerate FROG traces, we also measure the SHG FROG trace of the 1200 nm beam to validate the retrieved pulses from the cross-correlation FROG characterization. We measure the SHG signal, generated by two copies of the 1200 nm beam created by a beamsplitter, using a collinear autocorrelation FROG setup, as shown in Figure 1b.

In both optical measurement schemes, motorized delay stages are used to control the time delay between the two laser pulses before focusing the beams on a WS_2_ monolayer flake using a microscope objective (Olympus UPlanSapo) with a numerical aperture of 0.95. The WS_2_ monolayer is mechanically exfoliated from commercially available bulk WS_2_ onto a glass substrate (see also ref (15)). The emitted nonlinear signals from the WS_2_ monolayer are collected by the same objective and pass through a dichroic mirror and bandpass filters to filter the reflected fundamental light. The spectra of the nonlinear signals have been measured with a high-sensitivity cooled CCD-based spectrometer (SpectraPro 2300I). Figure 1c depicts a spectrum measured using the cross-correlation setup shown in Figure 1a with zero time delay between the fundamental pulses. This spectrum contains the nondegenerate signals of the SFG process around 470 nm, where ω_SFG_ = ω_775_ + ω_1200_, and FWM process around 572 nm, where ω_FWM_ = 2ω_775_ – ω_1200_. In addition, a peak can be seen around 600 nm originating from the second harmonic of the 1200 nm pulse, where ω_SHG_ = 2ω_1200_. These nonlinear signals have been well studied in WS_2_ and other 2D-layered materials.^14,15^ Finally, a broader peak at 625 nm is observed, which can be attributed to the PL signal of the WS_2_ monolayer. Note that the fundamental laser pulses with wavelengths of 775 nm and 1200 nm have a photon energy that is below the band gap of a WS_2_ monolayer. With these wavelengths, the band gap can only be excited via multiphoton excitation. Therefore, the PL signal observed here is emitted by nonlinearly generated excitons.

The nonlinear spectra are recorded for several time delays between the pulses to obtain the experimental FROG traces with an exposure time of 1 s. Here, we use an optical power of 2.4 mW for the 1200 nm laser beam and an optical power of 22.6 mW for the 775 nm beam that are below the damage threshold of the WS_2_ monolayer. The power dependency on the nonlinear response is more extensively described in Hernandez-Rueda et al.^15^ For both setups, the time delay is created by a motorized delay line in the 1200 nm beam path with spatial delay steps of 400 nm, equivalent to ∼1.3 fs. The spectrometer is calibrated by a rigid shift of the recorded spectrum. To reduce experimental noise, we fit a Gaussian shape over the nonlinear signals, and afterward, we interpolated the data onto a 1024 by 1024 matrix for the pulse retrieval algorithm.

**Figure 2:**
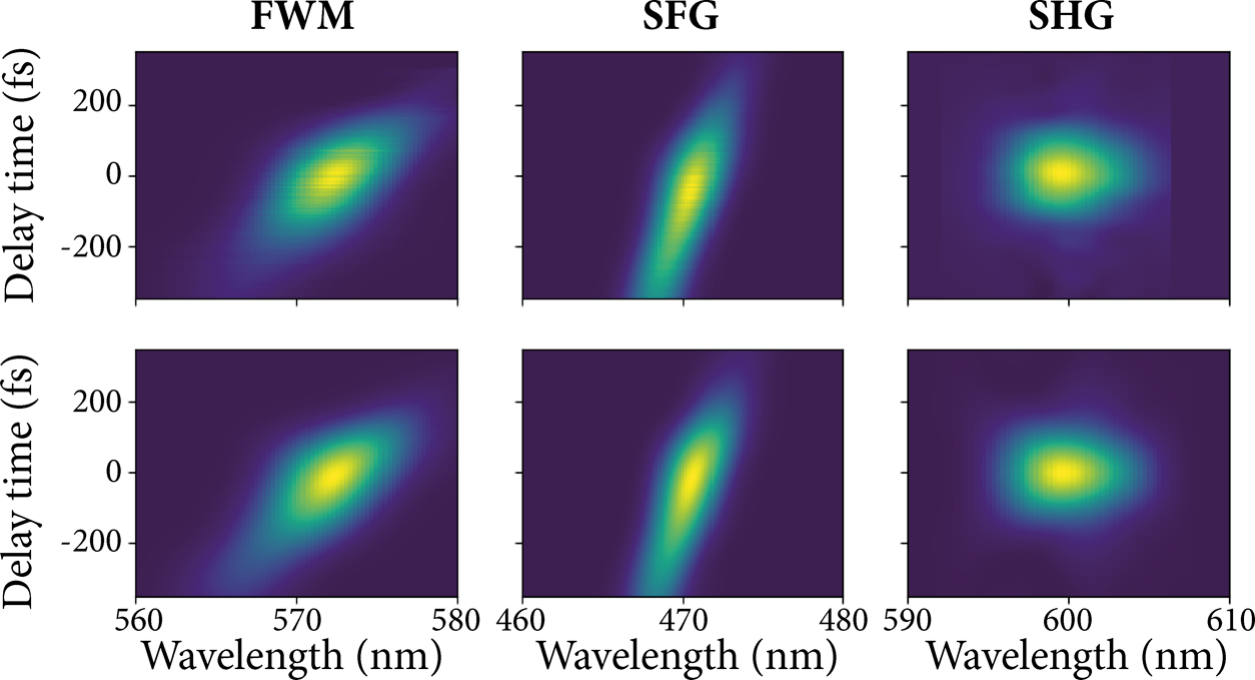
Top row displays the experimental FROG traces, where the FWM (left), SFG (middle), and SHG (right) signals are presented as a function of wavelength and time delay. The FWM and SFG FROG traces are simultaneously collected using the cross-correlation measurement scheme, shown in Figure 1a. The SHG spectra are collected using an autocorrelation setup (see Figure 1b). The bottom row displays the calculated FROG traces from the retrieved pulse shapes. For the FWM- and SFG-calculated FROG traces, both pulses were retrieved from the FWM and SFG measurement traces using an iterative cross-correlation FROG algorithm. For the SHG-calculated FROG trace, the 1200 nm pulse is retrieved from the SHG measurement trace using an autocorrelation FROG algorithm.

The top row of Figure 2 displays false color maps of the experimentally measured traces based on the FWM, SFG, and SHG nonlinear processes. On the *x*-axis, the wavelength of the nonlinear spectrum is plotted, and on the *y*-axis, the delay time between the two laser pulses is plotted. The top-left and middle panels depict the FWM and SFG traces, respectively, that are simultaneously collected from the same spectrum for each time delay using the optical setup sketched in Figure 1a. For both the experimentally measured FWM and SFG traces, an asymmetric pattern over the delay time can be observed. This asymmetric pattern indicates a frequency shift over the duration of at least one of the pulses, also referred to as a pulse chirp. Interestingly, a chirped pulse signal can be easily identified using nondegenerate FROG traces because the nonlinear frequency shifts over the delay time, while degenerate FROG traces are symmetric over the delay time and a chirped signal can only be deduced from the time–bandwidth product of the trace. The top-right panel in Figure 2 depicts a trace based on the SHG nonlinear process, which is measured independently using the optical setup sketched in Figure 1b. The SHG trace indeed shows a symmetric pattern over time delay.

## Results

The collected FROG traces of the SFG and FWM processes can now be used to extract the pulse intensity distribution and phase, *E*_1_ and *E*_2_, using the algorithm described in the Theory section. We initialize the pulse retrieval routine using two guess pulses with a Gaussian intensity distribution in the frequency domain, with a preset bandwidth of ∼ 16.7 nm full width at half-maximum (FWHM) and central wavelengths at 775 and 1200 nm. We first retrieve *E*_1_ with *E*_2_ as a reference pulse and then retrieve *E*_2_ using *E*_1_ as a reference pulse and iterate these two retrieval algorithms 15 times.

In Figure 2, the calculated SFG and FWM traces, generated using the retrieved pulse shapes, are presented below the experimental traces of the same processes. Good qualitative agreement can be observed between the measured and the calculated traces in Figure 2. To quantify the error of the retrieved FROG traces, we use a normalized root-mean-square trace error, *R*, that can be calculated from the measurement and retrieved traces. This normalized trace error is equivalent to the commonly used FROG error (see ref (22) for more details).

**Figure 3:**
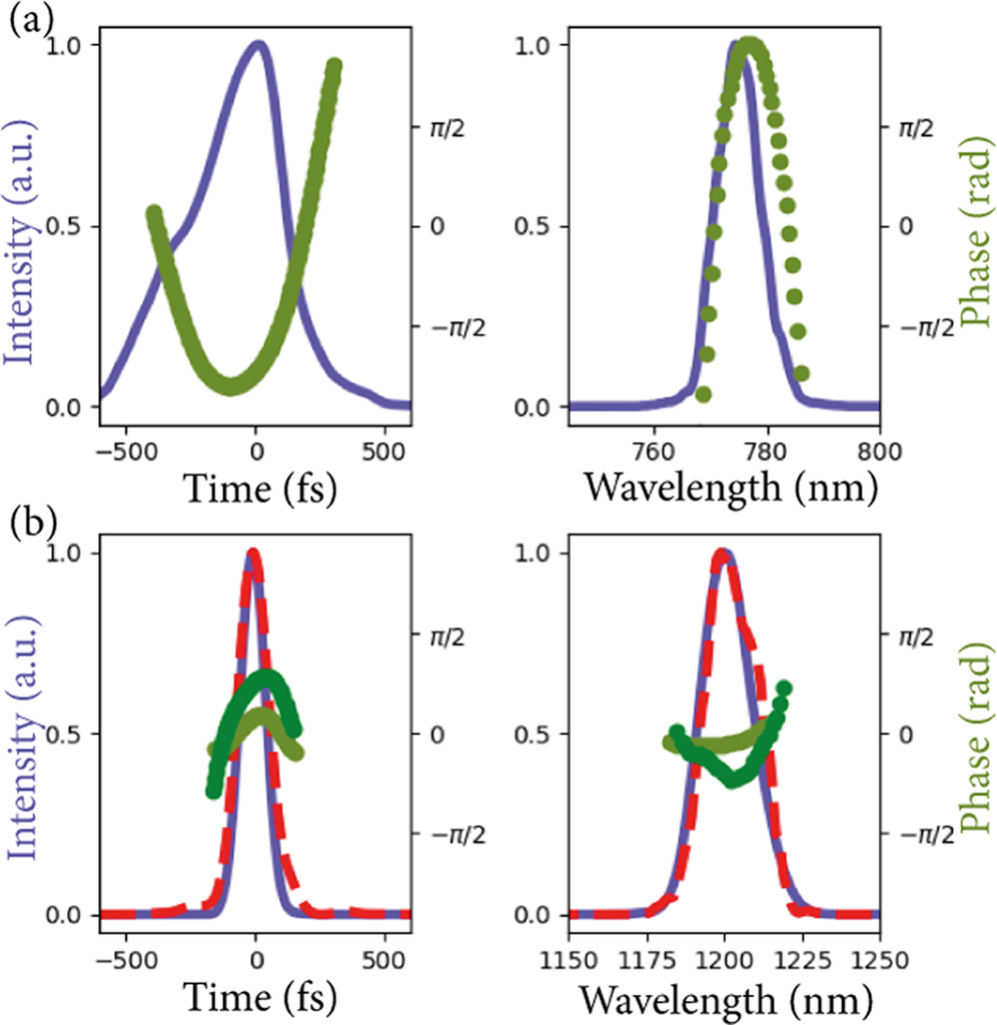
Retrieved temporal and spectral intensity distributions and phase of the ultrashort laser pulses at 775 nm (a) and 1200 nm (b). The pulse distributions plotted in purple (intensity) and light green (phase) are retrieved from the FWM and SFG measurement traces using an iterative cross-correlation FROG algorithm. The separately retrieved pulse distribution of the 1200 nm pulse from the SHG measurement trace using an autocorrelation FROG algorithm is plotted as a dashed red line (intensity) and dark green (phase).

The retrieved traces have a normalized error of 0.3% for both the FWM and SFG processes. In Figure 3, the retrieved complex temporal and spectral distributions of the two initial pulses are plotted. We observe a quadratic spectral phase signal in both the 775 and 1200 nm pulses, commonly referred to as chirp. In Figure 3a, we observe that the 775 nm pulse exhibits a more pronounced chirp. The chirp in both laser pulses is most likely caused by the various optical elements included in the optical setup and the laser system, i.e., the microscope objective that can be replaced by off-center parabolic mirrors to reduce the amount of chirp. We retrieve a longer pulse duration for the 775 nm pulse of 389 fs FWHM compared to the pulse duration of the 1200 nm of 115 fs FWHM.

To verify the validity of our results, we also measured a SHG FROG trace of the 1200 nm beam separately using the setup described in Figure 1b. The interference between the second harmonic of the two copies of the 1200 nm beams, caused by the collinear setup, is digitally filtered out to retrieve the FROG trace of a noncollinear setup, similar to the method employed by Janisch et al.^20^ Now, a noncollinear SHG FROG algorithm can be used to retrieve the pulse shape of the 1200 nm pulse. The right-most panels of Figure 2 depict the measured and retrieved traces of the SHG process. In Figure 3b, the temporal and spectral distributions of the intensity and phase of the retrieved 1200 nm pulse are presented. A good resemblance of the intensity distribution and phase between the retrieved pulses using SHG and SFG/FWM nonlinear signals is observed in Figure 3b. A retrieved pulse duration of 140 fs FWHM using SHG FROG is also similar to the 115 fs FWHM pulse duration obtained with the nondegenerate FROG measurement. In conclusion, we retrieve the pulse shapes of both laser pulses using the FWM and SFG FROG traces. The pulse shape of the 1200 nm beam determined in this way is reproduced with an independent FROG measurement using the SHG process in monolayer WS_2_.

## Nonlinear Photoluminescence

Nonlinear excitation pathways similar to those in SFG, SHG, and FWM mechanisms can mediate the generation of excitons through nonparametric processes. For frequencies above the band gap, light is absorbed; subsequently, the radiative recombination of the A-exciton also causes the characteristic photoluminescence signal at 625 nm, as observed in Figure 1c. Note that the band gap acts as an effective low-pass filter for our technique. The excitons can only be excited by multiphoton absorption (i.e., 2ω_1200_, 2ω_775_, ω_1200_ + ω_775_, 2ω_775_ – ω_1200_) because the photon energies at the fundamental wavelengths are smaller than the exciton band gap.^15,24,25^ The intensity of the photoluminescence therefore also changes with the time delay between the laser pulses due to the contribution of nondegenerate multiphoton excitation pathways, allowing for direct (cross-)correlation measurements.

**Figure 4:**
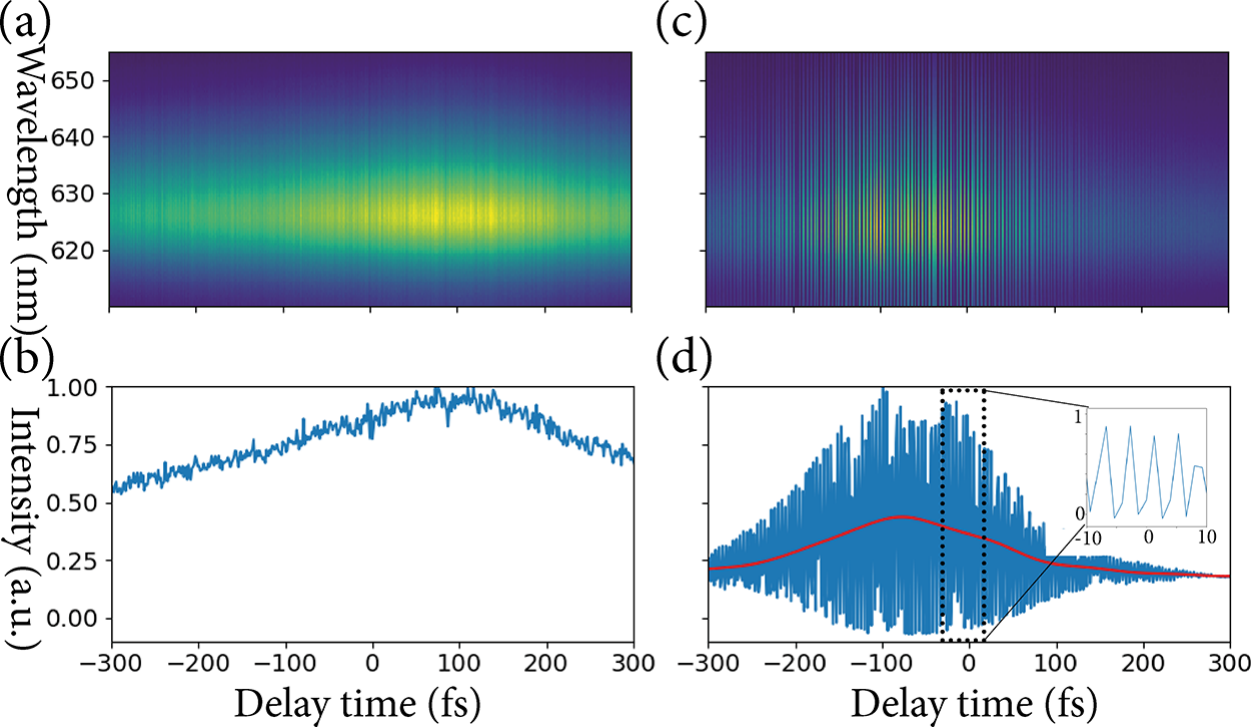
Experimental traces based on photoluminescence signals, where the PL signal is recorded using a nondegenerate measurement scheme (a) (see Figure 1a) and degenerate measurement scheme (c) (see Figure 1b). In panels (b) and (d), the projections over the wavelength axis are plotted for the measurement traces in panels (a) and (b), respectively. In panel (d), the interference pattern between the two 1200 nm beams is visible, where the DC component of the PL signal is plotted in red.

In Figure 4a, an experimental trace of the PL signal is shown, which is measured using the nondegenerate cross-correlation layout, where both 775 and 1200 nm beams are focused on the WS_2_ monolayer sample, as illustrated in Figure 1a. Figure 4b depicts the projection of the measurement trace in Figure 4a along the wavelength axis. The PL signal at zero time delay can be attributed to the generation of excitons from a combination of degenerate excitation pathways (i.e., 2ω_1200_, 2ω_775_) and nondegenerate excitation pathways (i.e., ω_1200_ + ω_775_, 2ω_775_ – ω_1200_). For long time delays, for which the individual pulses do not overlap in time, the PL signal is solely generated by degenerate multiphoton absorption mechanisms.

Figure 4c,d presents the PL measurement trace using the setup sketched in Figure 1b and its integration over the wavelength, where two sub-pulses of the 1200 nm beam are used. Because we use a collinear setup, the SHG measurement trace exhibits interference fringes, and since the excitons are now generated solely by the nonlinear processes, the fringes are also present in the PL signal (see Figure 4d). The period of these fringes agrees well with the 1200 nm wavelength of the initial laser beam. The DC component of the photoluminescence signal generated by the two-photon absorption is plotted as a red line in Figure 4d and can be understood as an effective SHG autocorrelation signal of the 1200 nm laser pulse. From the DC component, we extract a FWHM value, Δτ_*A*_^FWHM^, of 227 fs. This corresponds to a pulse duration, Δτ_*p*_^FWHM^, of 161 fs assuming a Gaussian pulse distribution of the 1200 nm pulse. This pulse duration is in close agreement with that extracted from the nondegenerate FROG method and the SHG FROG method.

## Conclusions

We have exploited the broad-band nonlinear response of an atomically thin WS_2_ monolayer due to its relaxed phase-matching conditions for ultrashort pulse characterization. Using a collinear optical setup, we measure both degenerate (SHG) and nondegenerate (FWM, SFG) nonlinear signals simultaneously by illuminating a WS_2_ monolayer with ultrashort laser pulses at two different wavelengths. We measure the nondegenerate nonlinear signals as a function of the time delay between the laser pulses to perform collinear (cross-) correlation FROG measurements. Using a novel adaptation of the COPRA FROG algorithm, we retrieve the complex pulse distribution of both laser pulses. An agreement is found between the retrieved pulses from the degenerate and nondegenerate FROG retrieval methods. We demonstrate the advantages of the simultaneous measurement of multiple nonlinear processes for ultrashort pulse characterization facilitated in 2D materials. In addition to the nonlinear parametric signals, we also measure the nonlinearly generated PL signal from the WS_2_ monolayer. The high nonlinear surface response over a broad range of wavelengths on an atomically thin TMDC allows for the implementation of novel retrieval methods simultaneously using multiple (non)degenerate nonlinear signals and PL emission, i.e., by also recording the spatial chirp of the nonlinear spectrum.^26^

## Supporting Information Available

Detailed derivation of the gradient steps for the SFG and FWM nonlinear processes (PDF)
