# Supplementary material for: Simultaneous Characterization of Two Ultrashort Optical Pulses at Different Frequencies Using a WS2 Monolayer
Source: ACS Photonics. 2022 May 10;9(6):1902–7. doi: 10.1021/acsphotonics.1c01270 (PMC9204806; doi:10.1021/acsphotonics.1c01270)
Supplement: Supplementary file 1 — ph1c01270_si_001.pdf [file ph1c01270_si_001.pdf]

Supplementary information: Simultaneous characterization of two ultrashort optical pulses at different frequencies using a WS_2 monolayer.

Marc Noordam, Javier Hernandez-Rueda,* and L. Kuipers*

Kavli Institute of Nanoscience Delft, Department of Quantum Nanoscience, Delft University of Technology, Lorentzweg 1, 2628 CJ Delft, The Netherlands

E-mail: fjavihr@gmail.com; L.Kuipers@tudelft.nl

1 Nondegenerate gradients

In the manuscript we use the common pulse retrieval algorithm (COPRA) algorithm developed by Geib et al.[1] to retrieve the fundamental pulse shapes from the FROG traces based on sum-frequency generation (SFG) and four-wave mixing (FWM). We extended the algorithm to also work with nondegenerate nonlinear signals and optimize for the SFG and FWM traces simultaneously. Here, we elaborate on the modification made with respect to the work of Geib et al.[1] For simplicity, we use the same formulation as the original paper. Here, $\tilde{E}_n(\omega)$ is the complex valued pulse envelope of the 775 nm and 1200 nm beams and $E_k(t)$ is its temporal counterpart. The time shifted 1200 nm pulse by the delay time τ is defined as,

$$A_{k,1200} = \mathcal{F}^{-1}[e^{i\tau\omega_{n,1200}}\tilde{E}_{n,1200}]. \tag{S1}$$

The spectral and temporal pulse shapes are related by following discrete Fourier transforms (DFT):

$$\tilde{E}_n = \mathcal{F}(E_k) \equiv \sum_k D_{nk}E_k \quad \text{with} \quad D_{nk} = \frac{\Delta t}{2\pi}e^{i\omega_n t_k} \tag{S2}$$

$$E_k = \mathcal{F}^{-1}(E_n) \equiv \sum_n D^{-1}_{kn}\tilde{E}_n \quad \text{with} \quad D^{-1}_{kn} = \Delta\omega e^{-i\omega_n t_k}. \tag{S3}$$

where Δt and $\Delta\omega$ are the time and frequency spacing respectively. Furthermore, the following relation between the DFT matrices is used:

$$D^{-1}_{kn} = \frac{2\pi\Delta\omega}{\Delta t}[D_{nk}]^*. \tag{S4}$$

The COPRA retrieval algorithm calculates for each iteration the distance,

$$Z = |S^{'} - S(\tilde{E})|^2 \tag{S5}$$

Where $S^{'}$ is the experimental FROG trace and $S(\tilde{E})$ is the calculated trace from the current solution $\tilde{E}$. $S(\tilde{E})$ for the nondegenerate nonlinear processes can be calculated in a similar

way as degenerate noncollinear FROG traces (even if the a collinear setup is used).

$$\mathcal{S}_k(\tilde{E}) = A_{k,1200}E_{k,775} = \mathcal{F}^{-1}[e^{i\tau\omega_{1200}}\tilde{E}_{n,1200}]\mathcal{F}^{-1}[\tilde{E}_{n,775}] \quad \text{(SFG)} \tag{S6}$$

$$\mathcal{S}_k(\tilde{E}) = A_{k,1200}E_{k,775}E_{k,775} = \mathcal{F}^{-1}[e^{i\tau\omega_{1200}}\tilde{E}_{n,1200}]\mathcal{F}^{-1}[\tilde{E}_{n,775}]\mathcal{F}^{-1}[\tilde{E}_{n,775}] \quad \text{(FWM)} \tag{S7}$$

Now, to retrieve the next iteration complex electric field a single-gradient descent step is performed. In our case we want to optimize for the both the FWM and the SFG measurement trace.

$$\nabla Z_{total} = \nabla(Z_{\text{FWM}} + Z_{\text{SFG}}) \tag{S8}$$

$$\nabla Z_{total} = \nabla Z_{\text{FWM}} + \nabla Z_{\text{SFG}} \tag{S9}$$

As discussed in section 3 of the supplement information of[1] the derivation of ∇Z goes as,

$$\nabla Z = 2\frac{\partial Z}{\partial \tilde{E}_n{}^*} \tag{S10}$$

$$\nabla Z = -2\sum_k \Delta S_k^* \frac{\partial S_k}{\partial \tilde{E}_n^*} + \Delta S(\frac{\partial S}{\partial \tilde{E}_n})^*. \tag{S11}$$

Here $\Delta S_k = S'_k - S_k(\tilde{E})$. Using the above formulas, we will derive the gradient steps for the SFG and FWM nonlinear processes. Note that we need to evaluate the gradient step for the two cases where we optimize the 1200 nm with the 775 nm as a reference and visa versa separately. So in total we derive four gradient steps: ∇Z_{SFG} for optimization of the 1200 nm pulse,∇Z_{SFG} for optimization of the 775 nm pulse, ∇Z_{FWM} for optimization of the 1200 nm pulse and ∇Z_{FWM} for optimization of the 775 nm pulse.

1.1 $\nabla Z_{\mathbf{SFG}}$ for optimizing 1200 nm beam

Combining equations S3 and S6 to calculate the partial derivatives of equation S11.

$$\frac{\partial S_k}{\partial \tilde{E}^*_{n,1200}} = 0 \tag{S12}$$

$$\frac{\partial S_k}{\partial \tilde{E}_{n,1200}} = \frac{\partial}{\partial \tilde{E}_{n,1200}} A_{k,1200} E_{k,775}$$

$$\frac{\partial S_k}{\partial \tilde{E}_{n,1200}} = E_{k,775} \frac{\partial}{\partial \tilde{E}_{n,1200}} \sum_l D^{-1}_{kl} e^{i\tau\omega_{l,1200}} \tilde{E}_{l,1200}$$

$$\frac{\partial S_k}{\partial \tilde{E}_{n,1200}} = D^{-1}_{kn} e^{i\tau\omega_{n,1200}} E_{k,775} \tag{S13}$$

Now we substitute equations S12 and S13 in equation S11,

$$\nabla Z_{\mathrm{SFG}} = -2 \sum_k \Delta S_k [D^{-1}_{kn} e^{i\tau\omega_{n,1200}} E_{k,775}]^*$$

$$\nabla Z_{\mathrm{SFG}} = -\frac{4\pi\Delta\omega}{\Delta t} e^{-i\tau\omega_{n,1200}} \sum_k D_{nk} \Delta S_{\tau,k} E^*_{k,775}$$

$$\boxed{\nabla Z_{\mathrm{SFG}} = -\frac{4\pi\Delta\omega}{\Delta t} e^{-i\tau\omega_{n,1200}} \mathcal{F}[\Delta S_{\tau,k} E^*_{k,775}]} \tag{S14}$$

1.2 $\nabla Z_{\mathbf{SFG}}$ for optimizing 775 nm beam

Combining equations S3 and S6 to calculate the partial derivatives of equation S11.

$$\frac{\partial S_k}{\partial \tilde{E}^*_{n,775}} = 0 \tag{S15}$$

$$\frac{\partial S_k}{\partial \tilde{E}_{n,775}} = \frac{\partial}{\partial \tilde{E}_{n,775}} A_{k,1200} E_{k,775}$$

$$\frac{\partial S_k}{\partial \tilde{E}_{n,775}} = A_{k,1200} \frac{\partial}{\partial \tilde{E}_{n,775}} \sum_l D^{-1}_{kl} \tilde{E}_{l,775}$$

$$\frac{\partial S_k}{\partial E_{n,775}} = D^{-1}_{kn} A_{k,1200} \tag{S16}$$

Now we substitute equations S15 and S16 in equation S11,

$$\nabla Z_{\text{SFG}} = -2 \sum_k \Delta S_k [D^{-1}_{kn} A_{k,1200}]^*$$

$$\nabla Z_{\text{SFG}} = -\frac{4\pi\Delta\omega}{\Delta t} \sum_k D_{nk} \Delta S_k A^*_{k,1200}$$

$$\boxed{\nabla Z_{\text{SFG}} = -\frac{4\pi\Delta\omega}{\Delta t} \mathcal{F}[\Delta S_k A^*_{k,1200}]} \tag{S17}$$

1.3 $\nabla Z_{\mathbf{FWM}}$ for optimizing 1200 nm beam

Combining equations S3 and S7 to calculate the partial derivatives of equation S11.

$$\frac{\partial S_k}{\partial \tilde{E}_{n,1200}} = 0 \tag{S18}$$

$$\frac{\partial S_k}{\partial \tilde{E}^*_{n,1200}} = \frac{\partial}{\partial \tilde{E}^*_{n,1200}} A^*_{k,1200} E_{k,775} E_{k,775}$$

$$\frac{\partial S_k}{\partial \tilde{E}_{n,1200}} = \frac{\partial}{\partial \tilde{E}_{n,1200}} \sum_l D^{-1*}_{kl} e^{-i\tau\omega_{l,1200}} \tilde{E}^*_{l,1200} E_{k,775} E_{k,775}$$

$$\frac{\partial S_k}{\partial \tilde{E}_{n,1200}} = D^{-1*}_{kn} e^{-i\tau\omega_{n,1200}} E_{k,775} E_{k,775} \tag{S19}$$

Now we substitute equations S18 and S19 in equation S11,

$$\nabla Z_{\text{FWM}} = -2 \sum_k \Delta S^*_k [D^{-1*}_{kn} e^{-i\tau\omega_{n,1200}} E_{k,775} E_{k,775}]$$

$$\nabla Z_{\text{FWM}} = -\frac{4\pi\Delta\omega}{\Delta t} e^{-i\tau\omega_{n,1200}} \sum_k D_{nk} \Delta S^*_k E_{k,775} E_{k,775}$$

$$\boxed{\nabla Z_{\text{FWM}} = -\frac{4\pi\Delta\omega}{\Delta t} e^{-i\tau\omega_{n,1200}} \mathcal{F}[\Delta S^*_k E_{k,775} E_{k,775}]} \tag{S20}$$

1.4 $\nabla Z_{\mathbf{FWM}}$ for optimizing 775 nm beam

Combining equations S3 and S7 to calculate the partial derivatives of equation S11.

$$\frac{\partial S_k}{\partial \tilde{E}^*_{n,775}} = 0 \tag{S21}$$

$$\frac{\partial S_k}{\partial \tilde{E}_{n,775}} = \frac{\partial}{\partial \tilde{E}_{n,775}} A^*_{k,1200} E_{k,775} E_{k,775}$$

$$\frac{\partial S_k}{\partial \tilde{E}_{n,775}} = \frac{\partial}{\partial \tilde{E}_{n,775}} A^*_{k,1200} \sum_l D^{-1}_{kl} \tilde{E}_{l,775} \sum_j D^{-1}_{kj} \tilde{E}_{j,775}$$

$$\frac{\partial S_k}{\partial \tilde{E}_{n,775}} = 2 D^{-1}_{kn} A^*_{k,1200} E_{k,775} \tag{S22}$$

Now we substitute equations S21 and S22 in equation S11,

$$\nabla Z_{\mathrm{FWM}} = -4 \sum_k \Delta S_k [D^{-1}_{kn} A^*_{k,1200} E_{k,775}]^*$$

$$\nabla Z_{\mathrm{FWM}} = -\frac{8\pi \Delta\omega}{\Delta t} \sum_k D_{nk} \Delta S_k A_{k,1200} E^*_{k,775}$$

$$\boxed{\nabla Z_{\mathrm{FWM}} = -\frac{8\pi \Delta\omega}{\Delta t} \mathcal{F}[\Delta S_{\tau,k} A_{k,1200} E^*_{k,775}]} \tag{S23}$$

References

(1) Geib, N. C.; Zilk, M.; Pertsch, T.; Eilenberger, F. Common pulse retrieval algorithm: a fast and universal method to retrieve ultrashort pulses. *Optica* **2019**, *6*, 495–505.
